# Thromboembolism in the Complications of Long COVID-19

**DOI:** 10.26502/fccm.92920317

**Published:** 2023-04-20

**Authors:** Leilani A Lopes, Devendra K Agrawal

**Affiliations:** 1 Western University of Health Sciences, College of Osteopathic Medicine of the Pacific – Northwest, Lebanon; 2 Western University of Health Sciences, College of Osteopathic Medicine of the Pacific, Pomona, CA

**Keywords:** COVID-19, D-dimers, Deep venous thrombosis, Inflammation, Long COVID-19, Stroke, Thromboembolism

## Abstract

SARS-CoV-2 is a +ssRNA helical coronavirus responsible for the global pandemic caused by coronavirus disease 19 (COVID-19). Classical clinical symptoms from primary COVID-19 when symptomatic include cough, fever, pneumonia or even ARDS; however, they are limited primarily to the respiratory system. Long-COVID-19 sequalae is responsible for many pathologies in almost every organ system and may be present in up to 30% of patients who have developed COVID-19. Our review focuses on how long-COVID-19 (3 –24 weeks after primary symptoms) may lead to an increased risk for stroke and thromboembolism. Patients who were found to be primarily at risk for thrombotic events included critically ill and immunocompromised patients. Additional risk factors for thromboembolism and stroke included diabetes, hypertension, respiratory and cardiovascular disease, and obesity. The etiology of how long-COVID-19 leads to a hypercoagulable state are yet to be definitively elucidated. However, anti-phospholipid antibodies and elevated D-dimer are present in many patients who develop thromboembolism. In addition, chronic upregulation and exhaustion of the immune system may lead to a pro-inflammatory and hypercoagulable state, increasing the likelihood for induction of thromboembolism or stroke. This article provides an up-to-date review on the proposed etiologies for thromboembolism and stroke in patients with long-COVID-19 and to assist health care providers in examining patients who may be at a higher risk for developing these pathologies.

## 1. Introduction

Coronavirus disease 19 (COVID-19) is a global pandemic caused by the severe acute respiratory syndrome coronavirus 2 (SARS-CoV-2), a positive sense RNA virus with a helical capsid [1]. The SARS-CoV-2 can enter into the body via several routes, including air droplets and ocular transmission [2]. The clinical symptoms from infection with a coronavirus typically presents as a spectrum from asymptomatic in mild cases, fever, cough, shortness of breath in moderate cases, and in severe cases may result in severe pneumonia, Acute Respiratory Distress Syndrome (ARDS), myocardial, ischemic, autonomic, and arrhythmogenic complications of COVID-19, and death [3,4]. Additionally, thrombotic events are common in patients with COVID-19, accounting for one third of COVID-19 patients in some studies [5–7].

Acute-COVID-19 is what is most associated with the clinical respiratory symptoms. However, long-COVID-19 or post-acute COVID-19 may occur in about 30% of patients between 3 – 24 weeks after resolution of the acute phase [8]. Sequelae from post-acute COVID-19 most commonly include respiratory (cough, dyspnea, and respiratory insufficiency) [9], myopathies (myalgia, fatigue, and malaise) [10] and circulatory symptoms, specifically thromboembolism with activation of inflammasomes [11,12]. Long term comlications of COVID-19 could also result in the development of tachycardia and atrial fibrillation, right heart dysfunction, myocardial stunning, and myocarditis-related fulminant heart failure, increased insulin resistance, fatigue, cognitive dysfunction, and increased neuroexcitability [13–15].

With COVID-19 constantly evolving, it has been difficult to extrapolate the relationship between COVID-19 and thrombotic events, specifically Arterial Thromboembolism (ATE) and Venous Thromboembolism (VTE) in patients who have acute- and long-term COVID-19. It is very likely that COVID-19 infection affects coagulation factors IX, XI, and XII and several immune cell functions resulting in atherothrombosis [16–18]. Thrombotic events for ATE could also be due to destabilization of atherosclerotic plaque resulting in limb arterial thrombosis, transient ischemic attack and stroke, cerebral infarction, and myocardial infarction [5,19,20]. VTE thrombotic events include Pulmonary Embolism (PE) and Deep Vein Thrombosis (DVT) [21,22]. Rates of thrombotic events in patients with COVID-19 differ depending on the type of study. In some post-mortem studies, prevalence of DVT was 58% [23]. However, a large cohort study of 40,254 patients showed that any relevant thrombotic event occurred in 7,113 patients (17.55%) where DVT specifically was in 1,761 patients (4.35%) [24]. Additionally, thrombotic events occurred both in hospital (acute COVID-19) and post-hospitalization (long-term COVID-19) [24].

More recently, evidence suggests that the different sequalae of disease is due to the immunological status of the patient rather than the virus itself, especially in patients with long-term COVID-19 [25]. Therefore, the underlying immunological etiologies and risk factors for thromboembolism in patients with COVID-19, especially long-term COVID-19, is critical for identifying COVID-19 patients who are more susceptible to thromboembolism and stroke and providing better care. This review will discuss the possible underlying etiologies of thromboembolism with COVID-19, risk factors associated with thromboembolism in COVID-19 patients, and the increased risk for stroke in patients with acute and long-term COVID-19.

## 2. Methods

In this comprehensive review, literature searches were conducted in PubMed and Google Scholar through December 2022. Key words included: thromboembolism COVID-19, post-acute COVID-19, long COVID-19, and DVT. Only articles in the English language were reviewed.

### 2.1 Etiologies of Thromboembolism

Although the underlying mechanism continues to evolve and has yet to be definitively elucidated, it has been postulated that the activation of the coagulation system contributes a major role to the adverse effects of COVID-19, specifically thromboembolism [6]. Additionally, due to some patients undergoing a cytokine storm, pro-inflammatory cytokines have thought to be involved in the immunologic response [26]. More recently, literature has suggested a correlation between the presence of antiphospholipid antibodies (aPLs) in COVID-19 patients who develop thromboembolism [21].

### 2.2 COVID-19-associated coagulopathy

COVID-19-Associated Coagulopathy (CAC) has been postulated as a new type of coagulopathy and shares many symptoms and factors with Disseminated Intravascular Coagulation (DIC), sepsis-induced coagulopathy, and many others [27]. It has been shown to induce PE in COVID-19 pneumonia patients and is especially prevalent in those in the Intensive Care Unit (ICU) [28]. Even with prophylactic treatment for thrombosis, the incidence of thrombotic patients of those with severe COVID-19 in the ICU was 31% in three Dutch hospitals [28].

An elevated D-dimer, the product of degradation of stabilized fibrin, has been shown to be increased in CAC, and elevated levels have been shown to be a predictor of disease prognosis [29–31]. However, this marker has limitations to assess the etiology of disease [30]. Additionally, there is a lack of data that exist as to whether patients with long-term COVID-19 have higher rates of CAC compared to patients with acute COVID-19.

### 2.3 Antiphospholipid antibodies

Antiphospholipid antibodies (aPLs) are autoantibodies that primarily target phospholipid-binding proteins and can contribute to antiphospholipid syndrome (APLS or APS) [32]. APLS is an autoimmune disorder characterized by thrombosis, early pregnancy, or fetal loss, and/or pregnancy morbidity with presence of persistently elevated aPLs [27,33].

aPLs have been associated with multiple infectious diseases such as retroviruses, herpesviruses, and some gram- positive and gram-negative bacteria [21,34]. Recently, aPLs have been found to be elevated in patients infected with SARS-CoV-2. The molecular mechanisms of the link between aPL and thromboembolism in patients with COVID-19 have yet to be elucidated. However, vascular endothelial damage inducing thrombin generation and excessive proinflammatory cytokines such as interleukins IL-1β and IL-6, TNFα, complement proteins, and coagulation factors among other immunologic mechanisms may produce activation of coagulopathies [16,21,35]. Further studies are needed to determine the extent of the link between aPLs and thromboembolism post-COVID-19 and the mechanisms by which thromboembolism is induced. In this regard, potential activation of inflammasomes, and adipokine dysregulation in the development of atherothrombosis and thromboembolism are the most likely events that warrant further investigation [11,13].

### 2.4 Inflammatory Cascade

SARS-CoV-2 binds to the hACE2-R receptor expressed in many tissues (eg. Lung, heart, liver, kidney and intestinal smooth muscle). Additionally, this receptor may also be found on the vascular endothelium and on immune cells. Upon binding of SARS-CoV-2, the hACE2-R is internalized, leading to accumulation of angiotensin II, upregulation of proinflammatory cytokines such as IL-1 and IL-6, reduction of IL-1β and a prothrombotic state [30,36,37].

In response to a virus, the human adaptive and innate immune system will usually release pro-inflammatory cytokines and a T-cell response. Overstimulation by viral antigen exhausts cytotoxic CD8+-T cells and eventually leads to secretion of CD279 (PD1) to signal for apoptosis [26,38]. Exhaustion markers such as NKG2A have been shown to be upregulated in patients with COVID-19, indicating the overstimulation of the T cell and NK cell response [39,40].

A chronic upregulation of proinflammatory cytokines and exhaustion of the immune system response place patients who suffer from long-COVID-19 in a prothrombotic state for a prolonged amount of time, increasing the risk for thromboembolism and systemic complications [36]

### 2.5 Comorbidities

Due to there being a possible link between the coagulation system and thromboembolism, patients who are in a hypercoagulable and hypofibrinolytic state, such as critically ill patients, are at a higher risk for developing thromboembolism [21]. Additionally, patients with long-COVID-19 may have symptomatology consistent with the presence of persistent plasma microthrombi that are resistant to fibrinolysis [41]

There are also many modifiable and non-modifiable risk factors that contribute to sequalae from acute COVID-19 such as cardiovascular disease, diabetes, obesity, and hypertension [40]. In long COVID-19, thromboembolism may be more prevalent in patients who are immunocompromised and more severely ill during their hospital stay [24,40]. Factors such as increased age, a Wells score of greater than 2 for DVT and greater than 6 for PE, and history of infection, cancer or VTE have been shown to increase the risk for developing thromboembolism during hospitalization [42]. Co-morbidities that may lead to increased risk for a thrombotic event post COVID-19 also include hypertension, diabetes mellitus, obstructive lung disease, chronic kidney disease and ischemic heart disease and thrombocytopenia [43].

Many of these risk factors are modifiable with lifestyle, where risk may be decreased before contracting acute COVID-19 and decreasing the risk of progression to long-COVID-19; however, this has yet to be understood. Due to the plethora of underlying pathologies that lead to a hypercoagulable state, it is vital to gain a deeper understanding into the most at-risk patients for thromboembolism to prophylactically treat patients before they develop post-COVID-19.

### 2.6 Long-COVID-19 and Stroke Risk

In addition to the increased risk for hematological pathologies such as DVT, PE and DIC, long-COVID-19 adverse risks include stroke [36]. The etiology behind long-COVID-19 induction of CNS effects is poorly understood. Many studies have reported an increased risk for patients, including children, with acute-COVID-19 developing cerebrovascular disease, specifically ischemic stroke, yet there is a lack of literature on the effects of long-COVID-19 in cerebrovascular phenomena [44].

It has been postulated that the interaction with SARS-CoV-2 and the ACE2 receptors within the Central Nervous System (CNS) can trigger a hypercoagulable and pro-inflammatory state via induction of vascular integrity disruption and the initiation of vasculitis. This in turn exposes the endothelium basement membrane and precipitates the activation of thrombosis. Due to the regulation of sympathoadrenal systems by ACE-2 receptors, SARS-CoV-2 interaction may disrupt intracranial and systemic blood pressure by downregulating autoregulation [45].

Long-COVID-19 has been shown to increase pro-inflammatory mediators, such as TNFα and IL-1, and lead to a hypercoagulable state. Typically, TNFα facilitates the maintenance of cerebral homeostasis and its excess may be present in conditions such as chronic conditions such as traumatic brain injury [46]. TNFα is produced by resident microglia and macrophages and has contrasting studies as to whether it is pathologic or protective in experimental stroke in mice [47]. IL-1, however, has been shown to be pathologic in experimental stroke [47]. These findings suggest that due to the upregulation of long-COVID-19 inflammatory markers, there may be an increased risk for cerebrovascular disease alike other chronic conditions [48–50]. Further studies are needed to determine the extent to which these pro-inflammatory mediators upregulated in long-COVID-19 are implicated in the pathogenesis of ischemic stroke.

## 3. Conclusion

SARS-CoV-2 leads to COVID-19, a worldwide pandemic, that has been implicated in many disease pathologies in almost every organ system. Importantly, the effects of COVID-19 persist even after acute infection in many patients, termed long-COVID-19 or post-COVID-19. One dangerous pathology from COVID-19 is thromboembolism due to COVID-19-associated coagulopathy (CAC) and may show an increased D-dimer and etiologies like DIC. Anti-phospholipid antibodies and upregulation of the inflammatory cascade in the vasculature and CNS are also present in patients with long-COVID-19 leading to a hypercoagulable state and placing the patient at risk for thromboembolism and stroke. Patients at a greater risk for developing these sequalae included critically ill and immunocompromised patients. Other risk factors included cardiovascular and lung disease, obesity, diabetes, and hypertension.

Studies on the potential mechanisms and risk factors are preliminary and further research is needed to understand the etiology of thromboembolism in long-COVID-19. However, this research provides a basis for patients who are at risk for developing thromboembolism, including stroke, post-COVID-19. These patients should be carefully monitored to reduce the risk of thromboembolism.

## Data Availability

Not applicable since the information is gathered from published articles.
